# New Insights into Pulmonary Hypertension: A Role for Connexin-Mediated Signalling

**DOI:** 10.3390/ijms23010379

**Published:** 2021-12-29

**Authors:** Myo Htet, Jane. E. Nally, Patricia. E. Martin, Yvonne Dempsie

**Affiliations:** 1 Department of Medicine, Division of Cardiology, Johns Hopkins University, Baltimore, MD 21205, USA; mhtet1@jhmi.edu; 2 School of Health and Life Sciences, Glasgow Caledonian University, Glasgow G4 0BA, UK; J.E.Nally@gcu.ac.uk (J.E.N.); Patricia.Martin@gcu.ac.uk (P.E.M.)

**Keywords:** pulmonary hypertension, connexins, gap junctions, vascular remodelling, hypoxic vasoconstriction, vascular reactivity, right ventricular hypertrophy

## Abstract

Pulmonary hypertension is a serious clinical condition characterised by increased pulmonary arterial pressure. This can lead to right ventricular failure which can be fatal. Connexins are gap junction-forming membrane proteins which serve to exchange small molecules of less than 1 kD between cells. Connexins can also form hemi-channels connecting the intracellular and extracellular environments. Hemi-channels can mediate adenosine triphosphate release and are involved in autocrine and paracrine signalling. Recently, our group and others have identified evidence that connexin-mediated signalling may be involved in the pathogenesis of pulmonary hypertension. In this review, we discuss the evidence that dysregulated connexin-mediated signalling is associated with pulmonary hypertension.

## 1. Introduction

### 1.1. Pulmonary Hypertension (PH)

Pulmonary hypertension (PH) is defined by a chronic increase in mean pulmonary arterial pressure [1]. The increase in pulmonary arterial pressure leads to right ventricular failure, which can be fatal. PH is associated with the narrowing of the distal pulmonary arteries due to pulmonary vascular remodelling [2]. Pulmonary artery endothelial cells (PAECs), pulmonary artery smooth muscle cells (PASMCs) and pulmonary artery fibroblasts (PAFs) are all involved in the pulmonary vascular remodelling process. The medial layer of the pulmonary artery (composed predominantly of PASMCs) becomes thickened. The intimal layer can also become thickened, and disorganised proliferation of intimal PAECs leads to the formation of plexiform lesions. Obliterative concentric lesions, characterised by an onion skin arrangement of PAECs and/or PASMCs are also seen in patients with PH [2]. PAFs derived from patients with PH and from animal models of PH have been shown to have a hyperproliferative, apoptosis-resistant and pro-inflammatory phenotype. In addition, PAFs have been shown to induce proliferation and migration of PASMCs through the release of mitogens [3,4,5].

The increase in mean pulmonary arterial pressure observed in patients with PH can be due to a variety of causes, thus PH can be sub-divided into five main clinical groups (Table 1): pulmonary arterial hypertension (PAH; group 1), PH due to left heart disease (group 2), PH due to chronic lung diseases or hypoxia (group 3), chronic thromboembolic PH (group 4) and PH with unclear multifactorial mechanisms (group 5). PAH can be idiopathic (for which there is no known cause), heritable (commonly associated with mutations in the bone morphogenetic protein receptor type 2) or associated with the ingestion of certain drugs such as dexfenfluramine and methamphetamine. PAH can also be associated with certain conditions such as connective tissue disease or HIV infection [6]. Throughout this review, we will discuss PAH and PH due to chronic lung disease or hypoxia. Animal models will be referred to as models of PH.

Despite recent advances in our understanding of PH, current therapies serve only to prolong life and increase the quality of life. They are unable to reverse disease progression, and prognosis remains poor. In addition, current available therapies are primarily targeted at PAH. The management of group 2–5 PH focusses on treating the underlying disorder [7]. Therefore, there is an urgent need for the development of novel therapeutic agents with which to treat PH.

### 1.2. Overview of Connexins

**Figure 1:**
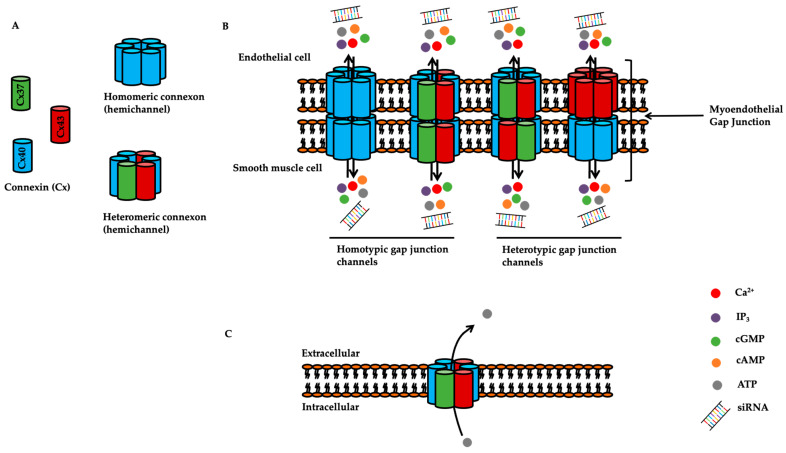
Structure of hemi-channels and gap junctions. (**A**) Six connexins assemble to form a homomeric or heteromeric connexon (also known as hemi-channel). (**B**) Two connexons from adjacent cells dock with each other to form a gap junction (homotypic or heterotypic) which allows the exchange of molecules less than 1 kD in molecular weight such as Ca^2+^ (calcium), IP_3_ (inositol triphosphate), cGMP (cyclic guanosine monophosphate), cAMP (cyclic adenosine monophosphate), ATP (adenosine triphosphate) and siRNA (small interfering RNA). (**C**) The connexon can also permit the release of molecules such as ATP (adenosine triphosphate) from the intracellular to the extracellular space.

Connexins are transmembrane proteins which can assemble to form gap junctions between cells for the exchange of small molecules, less than 1 kD in molecular weight. In addition, they can also form hemi-channels or connexons in the plasma membrane for adenosine triphosphate (ATP) release and subsequent autocrine and/or paracrine signalling (Figure 1). The half-life of connexins can range from 1.5 to 5 h [8,9]. There are 20 connexin genes in mouse and 21 in human. According to sequence homology and length of cytoplasmic loops, these connexins can be grouped into alpha (α), beta (β), gamma (γ), delta (δ) and zeta (ζ) [10,11]. Structurally, each connexin possesses four domains (M1, M2, M3 and M4) spanning across the plasma membrane, with two extracellular loops (E1 and E2) and one intracellular loop (IL) and amino (NH_2_) and carboxyl (COOH) termini facing the cytoplasm. Each extracellular loop contains three highly conserved cysteine residues. Six connexins are required to oligomerize to form a connexon (Figure 1). The connexon oligomerization can be patterned as homomeric (composed of identical connexins) or heteromeric (composed of different connexins). Disulphide bridges formed between the cysteine residue of the two extracellular loops help maintain structural integrity and dock two opposing connexons [12,13]. Docking of two connexons from adjacent cells forms a gap junction (Figure 1). The formed gap junctions can also be composed of homotypic or heterotypic connexons. Collectively, patterns of gap junctions can be homomeric homotypic, heteromeric homotypic, homomeric heterotypic or heteromeric heterotypic [14,15,16]. Classically, gap junctions formed by connexins permit the transfer and exchange of small molecules less than 1 kD in molecular weight, including but not limited to, inositol triphosphate (IP_3_), cyclic adenosine monophosphate (cAMP), cyclic guanosine monophosphate (cGMP), adenosine trisphosphate (ATP) and even small interfering RNAs (siRNAs) [12,17,18]. In addition to this, connexins have also been shown to be present in exosomes and to be involved in communication between exosomes and their target cells [19].

## 2. Expression and Function of Connexins in the Pulmonary Circulation

### 2.1. Expression of Connexins in the Pulmonary Vasculature

Within the pulmonary vasculature, studies have confirmed that Cx37, Cx40, Cx43 and Cx45 are expressed (Table 2). Studies in rat pulmonary arteries determined that Cx43 protein was localised mainly in the endothelium, whereas Cx40 and Cx37 proteins were detected in both endothelial and smooth muscle layers [20,21,22]. Gene expression of Cxs 37, 40, 43 and 45 has been shown in rat PASMCs and PAFs [23,24]. Evidence has confirmed that rat pulmonary artery endothelial gap junctions (gap junctions formed between endothelial cells) consist of Cx43, Cx40 and Cx37; however, they are formed primarily by Cx43 and Cx40 [25,26]. In vitro studies showed that the myoendothelial gap junctions (MEJ) between rat PAECs and PASMCs were primarily formed by Cx43 [27]. Among the pulmonary vascular connexins, we recently reported that the Cx43 gene (*GJA1*) was the predominant connexin gene expressed in both rat PAFs and PASMCs [24]. Studies on the role of connexins in the regulation of the pulmonary vasculature have so far focused on Cx40 and Cx43, with some information available on Cx37. The role of Cx45 in the regulation of the pulmonary vasculature has yet to be elucidated.

### 2.2. Altered Connexin Expression in the Pulmonary Vasculature of Patients with PH and Animal Models of PH

Cx43 protein expression was increased in pulmonary arteries from patients with chronic hypoxic PH and decreased in PASMCs derived from patients with idiopathic PAH. There was no change in Cx43 protein expression in PAECs derived from patients with idiopathic PAH [28]. This suggests Cx43 may have a cell type-specific role. Interestingly, these data also suggest Cx43 may have a distinct role in idiopathic PAH compared to chronic hypoxic PH, and further investigations into this are warranted.

Expression of Cxs 37 and 40 has also been shown to be dysregulated in patients with PAH [29]. Kim and colleagues determined that Cx37 and Cx40 protein expression was reduced in PAECs derived from PAH patients. In addition, Cx37 and Cx40 expression was mediated by transcription factor myocyte enhancer factor 2 (MEF2), and its transcriptional activity was impaired in PAH [29]. The expression of MEF2 was regulated by phosphorylation and cytoplasmic translocation of histone deacetylases (HDACs) HDAC4 and HDAC5 [30]. The protein expression of HDAC4 and HDAC5 was increased in the lungs of PAH patients [31]. Augmentation of MEF2 by siRNA inhibition of HDAC4 and HDAC5 increased Cx37 and Cx40 expression in PAECs from PAH patients and promoted disease rescue in the monocrotaline rat model [29]. In addition, Cx40 expression was decreased in PAECs from mice with chronic hypoxia-induced PH [32] as well as in PASMCs from monocrotaline rats [33].

Hypoxia causes pulmonary vasoconstriction and pulmonary vascular remodelling and therefore can lead to the development of PH, [34]. Due to this, hypoxia is a commonly used model of PH. Chronic hypoxia has been shown to increase Cx43 gene expression in rat pulmonary arteries [21]. In line with this, our group found acute hypoxic exposure for 24h increased Cx43 protein expression in rat PAFs. On the other hand, we found Cx43 gene expression to be downregulated in pulmonary arteries derived from mice exposed to two weeks of chronic hypoxia [35]. In vitro experiments also determined that Cx43 protein expression was downregulated in rat PASMCs in response to acute hypoxia for 24h [36]. Discrepancies between these studies may be due to the cell/tissue type studied, hypoxic duration, and oxygen concentration. Changes in protein expression of connexins in patients with PH and in animal models of PH is summarised in Table 3.

### 2.3. Oestrogen-Induced Regulation of Connexin Expression

One of the major risk factors for the development of PAH is female sex. Female adults under ~60 years old are around three times more likely than males of a similar age to develop PAH; however, this sex difference does not persist in the older population [37]. This female bias has been associated with the sex hormone oestrogen [38]. It is therefore of interest that oestrogen can regulate the expression of various connexins [39]. For example, oestrogen has been shown to upregulate Cx43 gene and protein expression in human myometrium [40,41], osteocyte-like MLO-Y4 cells [42] and rat myocardium [43]. In addition, inhibition of oestrogen receptors with fulvestrant reduced Cx43 gene and protein expression in breast cancer cells [44]. Multiple oestrogen response elements have been identified in the Cx43 promoter. It is thought that transcription factor activator protein 1 (AP-1) is involved in oestrogen-induced up-regulation of Cx43 [40]. In line with these findings, we have shown Cx43 gene expression to be up-regulated in pulmonary arteries from female mice compared to male mice [35].

### 2.4. Connexin-Mediated Signalling in Pulmonary Vascular Reactivity

Connexins have been shown to be involved in serotonin- (5-HT) and endothelin- (ET-1) induced contraction of the pulmonary vasculature. It has been well established that both 5-HT and endothelin signalling is implicated in the pathogenesis of PH [45,46]. Billaud and colleagues determined that 5-HT-induced vasoconstriction in isolated rat pulmonary arteries was inhibited by the connexin mimetic peptide (^37,43^Gap27) which inhibits Cx37 and Cx43 [21]. Furthermore, reactive oxygen species (ROS) produced in the pulmonary artery smooth muscle cells upon 5-HT-induced contraction can travel back to the endothelial cells through MEJ and scavenge the vasodilator molecule NO [20]. We and others have found that ET-1-induced contraction is increased in intra-lobar pulmonary arteries derived from Cx43 heterozygous mice compared to wildtype mice [28,35].

Connexins have also been shown to be involved in pulmonary vasodilator responses. Methacholine-induced pulmonary vasodilation was reduced in the presence of ^37,43^Gap27. Methacholine-induced pulmonary vasodilation was also reduced in intralobar pulmonary arteries derived from Cx43 heterozygous knockout mice (Cx43^+/−^ mice) compared to wildtype mice [35]. Interestingly, a recent study found Cx43 is a target of mi-R1, and incubation with mi-R1 decreased acetylcholine-induced vasodilatory responses in PA, which was associated with a reduction in Cx43 expression and an increase in O_2_^-^ production [47]. It has been well established that mi-R1 is increased in hypoxia-induced PH [48,49]. Whether the reduction in Cx43 expression observed in some studies after exposure to hypoxia [35,36] is a direct effect of hypoxia exposure or an indirect effect through the induction of mi-R1-dependent Cx43 mRNA degradation is worthy of investigation.

Within the pulmonary vasculature, hypoxia plays an important role in mediating pulmonary vasoconstriction. The mechanisms involved in hypoxic pulmonary vasoconstriction are complex and poorly understood. Multiple mechanisms such as dysregulated potassium channel expression, abnormal Ca^2+^ entry and release mechanisms and processes involving reactive oxygen species and mitochondria have been proposed in the past [50,51,52]. There is also strong evidence to support a role for Cx40 in hypoxic pulmonary vasoconstriction. Hypoxic pulmonary vasoconstriction was reduced in isolated perfused lungs of Cx40 knock-out (Cx40^−/−^) mice compared to wildtype mice. Hypoxic vasoconstriction was also attenuated in the presence of the non-specific gap junction blocker 18β-glycyrrhetinic acid (18β-GA) or ^40^Gap27, a Cx40-specific blocker [53]. Cx43 immunostaining, protein expression and phosphorylation levels did not differ between Cx40^+/+^ and Cx40^−/−^ mouse lungs, suggesting functional gap junctions were present in Cx40^−/−^ mice. Interestingly, there was an additive effect in attenuating hypoxic vasoconstriction by a combination of ^40^Gap27 and ^43^Gap27, which is a Cx43-specific blocker [53]. This suggests Cx43 may also be involved in the process of hypoxic vasoconstriction. Another group later showed nonspecific gap junction inhibitors such as 18β-GA, heptanol and 2-aminoethoxydiphenyl borate (2-APB) abolished the sustained phase of hypoxic pulmonary vasoconstriction produced by prostaglandin F2 alpha (PGF2α) in isolated rat intralobar pulmonary arteries without affecting the intracellular Ca^2+^ concentration. Subsequently, it was confirmed that 18β-GA attenuated hypoxic pulmonary vasoconstriction via inhibition of Rho kinase-dependent Ca^2+^ sensitization [54]. This suggests gap junctions may play a role in intracellular calcium sensitization during hypoxic vasoconstriction processes. In line with a role for Cx40 in pulmonary vascular reactivity, a recent study has shown that Cx40 plays a role in endothelium-dependent hyperpolarisation (EDH)-mediated relaxation in mouse small distal pulmonary arteries. The authors have further shown that in mice, chronic hypoxia decreases endothelial Cx40 and therefore attenuates EDH-mediated vasodilation, contributing to the development of PH [32].

### 2.5. Connexin-Mediated Signalling in Pulmonary Vascular Remodelling and Development of PH

Cx43^+/−^ mice have been shown to be protected against hypoxic-induced pulmonary vascular remodelling and lung inflammation. However, hypoxia-induced increases in right ventricular systolic pressure or right ventricular hypertrophy were similar in Cx43^+/−^ mice compared to wildtype mice [28]. Interestingly, Cx43 has been shown to be involved in hypoxia-induced proliferation and migration of rat PAFs, which may lead to pulmonary vascular remodelling. Hypoxia-induced proliferation and migration of rat PAFs were inhibited pharmacologically by ^37,43^Gap27 and also by genetic reduction of Cx43 using an siRNA approach [24]. In addition, ^37,43^Gap27 reduced hypoxic-induced phosphorylation of ERK1/2 and p38 MAP kinase, both of which have been shown to play a role in hypoxia-induced proliferation and migration of rat PAFs. It is important to note, however, that (as discussed above) dysregulated expression of vascular connexins in patients with PH may be specific to a subgroup of PH. For example, Cx43 expression is decreased in PASMCs from patients with PAH, while it is increased in pulmonary arteries from patients with PH associated with hypoxia. Therefore, as well as assessing the role of connexins in hypoxic models of PH, it will also be important to assess the role of connexins in the development of PAH in vivo using suitable animal models such as the SUGEN/hypoxic model. In addition to data derived from hypoxic models, it has been shown that Cx43 is important in serotonin signalling in the pulmonary vasculature. 5-HT is synthesised and released from PAECs and acts on neighbouring PASMCs to promote their differentiation and proliferation [55,56]. It has been shown that 5-HT passes from rat PAECs to rat PASMCs through MEJ, as the transfer of 5-HT between these cell types could be inhibited by the non-specific gap junction blocker carbenoxolone or by siRNA knockdown of Cx43 [56].

Cx40^−/−^ mice have also been shown to be protected against hypoxia-induced pulmonary vascular remodelling. However, unlike Cx43 heterozygous mice, Cx40^−/−^ mice were protected against hypoxia-induced increases in right ventricular systolic pressure and right ventricular hypertrophy [53]. As discussed above, Cx40 is thought to be important in hypoxic pulmonary vasoconstriction; it may occur, through the inhibition of hypoxic pulmonary vasoconstriction, that Cx40^−/−^ mice are protected against hypoxia-induced PH. The investigation of the possible role of Cx40 in the proliferation of pulmonary vascular cells would be of interest.

Post-translational modification of connexin proteins can influence the proliferation of vascular cells [57]. For example, in systemic vascular smooth muscle cells, MAPK-phosphorylated Cx43 has been shown to interact with cyclin E to enhance proliferation [58]. On the other hand, post-translational modification of Cx37 has been associated with growth-suppressive effects in a variety of cell lines [59,60,61]. In the pulmonary vasculature, a recent study showed that hypoxia caused phosphorylation of Cx43 in rat pulmonary arteries and rat PASMCs and that hypoxia-induced proliferation of PASMCs was inhibited by ^37,43^Gap27 or the knockdown of Cx43 [62]. As of yet, however, the effects of post-translational modifications of connexins on the proliferation of pulmonary vascular cells remain to be investigated.

### 2.6. Connexin-Mediated Signalling in the Right Ventricle in Animal Models of PH

Cx43 is abundantly expressed in the heart; Cx40 and Cx45 are also expressed, although in lower quantities than Cx43. Aberrant connexin expression has been linked to a variety of cardiac disorders [63]. With regard to PH, multiple studies have shown that Cx43 protein expression was downregulated in the right ventricle of the monocrotaline rat model [64,65,66]. In addition to this, in hypertrophic right ventricles of monocrotaline rats, gap junctions that were immunolabelled with Cx43 were internalized [67]. Interestingly, another study showed that the treatment with a dual endothelin receptor antagonist improved the redistribution of Cx43 in the myocardium of right ventricles derived from monocrotaline rats [68]. In addition, intra-tracheal gene delivery of sarcoplasmic/endoplasmic reticulum Ca^2+^ ATPase 2a (SERCA2a) in monocrotaline-treated rats restored cardiac Cx43 gene and protein expression and improved ventricular tachycardia [69]. However, in vivo functional studies investigating the role of Cx43 in right ventricular failure associated with PH are lacking. One study has shown that hypoxia-induced right ventricular hypertrophy was unchanged in Cx43 heterozygous mice compared to wildtype mice [28]. A thorough investigation into the role of Cx43 in right ventricular failure associated with PH is warranted.

**Table 3:** Summary of the changes in protein expression of Cx37, Cx40, Cx43 and Cx45 in patients with PH and in animal models of PH. Summary of the role of Cx37, Cx40, Cx43 and Cx45 in the development of PH in animal models. ↔ no change, ↑ increased, ↓ decreased.

Connexins	Protein Expression in PH/PAH Patients	Protein Expression in Animal and Cellular Models of PH	Role in Development of PH in Animal Models
Cx37	↓ in PAECs from PAH patients [29]	↔ in rat PASMCs exposed to acute hypoxia [24] ↑ in rat PAFs exposed to acute hypoxia [24] ↓ in human lung tissue section from PAH patients [29]	Unknown
Cx40	↓in PAECs from PAH patients [29]	↓ protein expression in PAECs from mouse with chronic hypoxia-induced PH [32] ↓ protein expression in rat PASMCs exposed to acute hypoxia [24] ↔ protein expression in rat PAFs exposed to acute hypoxia [24] ↓ protein expression in lung tissues from the rat monocrotaline model [33]	Hypoxic pulmonary vasoconstriction reduced in Cx40^−/−^ mice and by pharmacological inhibition of Cx40 [53] Cx40^−/−^ mice are protected against hypoxia-induced PH [53]
Cx43	↑in PAs from patients with chronic hypoxic PH [28] ↓in PASMCs in patients with idiopathic PAH [28] ↔ in PAECs in patients with idiopathic PAH [28]	↓ in whole lung tissue from chronic hypoxia mouse [35] ↑ in whole lung tissue from sugen /hypoxic rat [24] ↑ in rat PAFs exposed to acute hypoxia [24] ↔ in rat PASMCs exposed to acute hypoxia (5% O_2_) [24] ↓ in rat PASMCs exposed to acute hypoxia (3% O_2_) [36] ↓ in right ventricle of rat monocrotaline model [64,65,66,69] Internalization and lateralization of Cx43 in the right ventricle of the rat monocrotaline model [67,68]	Cx43^+/−^ mice are protected against hypoxia-induced pulmonary vascular remodelling and lung inflammation [28]
Cx45	Unknown	↔ in response to acute hypoxia in rat PASMCs and rat PAFs [24]	Unknown

## 3. Conclusions

The majority of studies have assessed the role of Cx40 and Cx43 in the regulation of the pulmonary vasculature, with little being known about the function of Cx37 and Cx45 (Table 3). Cellular communication via Cx40 and Cx43 plays a role in pulmonary vascular reactivity, while Cx43 has also been shown to be involved in pulmonary vascular cell proliferation. Abnormal pulmonary vasoreactivity and cellular proliferation can lead to the pulmonary vascular contraction and remodelling associated with PH. Indeed, both Cx40 and Cx43 have been shown to be involved in the development of murine hypoxia-induced PH. It is possible that dysregulation of connexin expression differs between subgroups of PH, and therefore, it is also necessary to assess whether connexins are involved in the development of PAH using suitable animal models.

Ultimately, more research is required to elucidate whether targeting aberrant connexin function may be a novel therapeutic strategy for PH. Currently available drugs which target specific connexins are peptides. The reduced bioavailability of peptide drugs presents problems for both pre-clinical and translational research. Current in vivo studies on the role of connexins in the development of PH have been conducted on genetically modified mice. Currently, there are no published studies assessing the effects of the pharmacological targeting of connexins on the development of PH in animal models. However, much research is currently ongoing to improve the administration and the bioavailability of these drugs, for example, using exosomes as vehicles for peptide delivery [70]. Indeed, connexin mimetic peptides and analogues thereof are receiving increased attention in translational research in areas related to cardiovascular disease, cancer, neurological disorders and wound healing [71].

## Figures and Tables

**Table 1:** Classification of pulmonary hypertension (PH).

Group 1	Pulmonary arterial hypertension (PAH) Idiopathic PAH Heritable PAH Drug and toxin induced Associated with Connective tissue disease HIV infection Portal hypertension Congenital heart diseases Schitosomiasis Pulmonary veno-occlusive disease Persistent pulmonary hypertension of the newborn
Group 2	PH due to left heart disease
Group 3	PH due to lung disease and/or hypoxia
Group 4	Chronic thromboembolic PH (CTEPH)
Group 5	PH with unclear or multifactorial mechanisms

**Table 2:** Expression of connexins in cells of the pulmonary vasculature.

Cell Type	Connexins	References
Pulmonary artery endothelial cells (PAECs)	Cx43, Cx40, and Cx37	[20,21,22,25,26,27]
Pulmonary artery smooth muscle cells (PASMCs)	Cx43, Cx40, Cx37, and Cx45	[20,21,22,23,25,26,27]
Pulmonary artery fibroblasts (PAFs)	Cx43, Cx40, Cx37, and Cx45	[24]

## References

[1] Simonneau G., Montani D., Celermajer D.S., Denton C.P., Gatzoulis M.A., Krowka M., Williams P.G., Souza R. (2019). Haemodynamic definitions and updated clinical classification of pulmonary hypertension. Eur. Respir. J..

[2] Tuder R.M. (2017). Pulmonary vascular remodeling in pulmonary hypertension. Cell Tissue Res..

[3] Zhang H., Wang D., Li M., Plecitá-Hlavatá L., D’Alessandro A., Tauber J., Riddle S., Kumar S., Flockton A., McKeon B.A. (2017). Metabolic and proliferative state of vascular adventitial fibroblasts in pulmonary hypertension is regulated through a microRNA-124/PTBP1 (polypyrimidine tract binding protein 1)/pyruvate kinase muscle axis. Circulation.

[4] Carlin C.M., Celnik D.F., Pak O., Wadsworth R., Peacock A.J., Welsh D.J. (2012). Low-dose fluvastatin reverses the hypoxic pulmonary adventitial fibroblast phenotype in experimental pulmonary hypertension. Am. J. Respir. Cell Mol. Biol..

[5] Wilson K.S., Buist H., Suveizdyte K., Liles J.T., Budas G.R., Hughes C., MacLean M.R., Johnson M., Church A.C., Peacock A.J. (2020). Apoptosis signal-regulating kinase 1 inhibition in in vivo and in vitro models of pulmonary hypertension. Pulm. Circ..

[6] Simonneau G., Gatzoulis M.A., Adatia I., Celermajer D., Denton C., Ghofrani A., Gomez Sanchez M.A., Krishna Kumar R., Landzberg M., Machado R.F. (2013). Updated clinical classification of pulmonary hypertension. J. Am. Coll. Cardiol..

[7] Sysol J.R., Machado R.F. (2018). Classification and pathophysiology of pulmonary hypertension. Contin. Cardiol. Educ..

[8] Fallon R.F., Goodenough D.A. (1981). Five-hour half-life of mouse liver gap-junction protein. J. Cell Biol..

[9] Beardslee M.A., Laing J.G., Beyer E.C., Saffitz J.E. (1998). Rapid turnover of connexin43 in the adult rat heart. Circ. Res..

[10] Nielsen M.S., Axelsen L.N., Sorgen P.L., Verma V., Delmar M., Holstein-Rathlou N.H. (2012). Gap junctions. Compr. Physiol..

[11] Abascal F., Zardoya R. (2013). Evolutionary analyses of gap junction protein families. Biochim. Biophys. Acta (BBA) Biomembr..

[12] Martin P.E., Evans W.H. (2004). Incorporation of connexins into plasma membranes and gap junctions. Cardiovasc. Res..

[13] Bai D., Yue B., Aoyama H. (2018). Crucial motifs and residues in the extracellular loops influence the formation and specificity of connexin docking. Biochim. Biophys. Acta (BBA)-Biomembr..

[14] Söhl G., Willecke K. (2004). Gap junctions and the connexin protein family. Cardiovasc. Res..

[15] Kumar N.M., Gilula N.B. (1996). The gap junction communication channel. Cell.

[16] Evans W.H., Martin P.E. (2002). Gap junctions: Structure and function. Mol. Membr. Biol..

[17] Meşe G., Richard G., White T.W. (2007). Gap junctions: Basic structure and function. J. Investig. Dermatol..

[18] Valiunas V., Polosina Y.Y., Miller H., Potapova I.A., Valiuniene L., Doronin S., Mathias R.T., Robinson R.B., Rosen M.R., Cohen I.S. (2005). Connexin-specific cell-to-cell transfer of short interfering RNA by gap junctions. J. Physiol..

[19] Soares A.R., Martins-Marques T., Ribeiro-Rodrigues T., Ferreira J.V., Catarino S., Pinho M.J., Zuzarte M., Anjo S.I., Manadas B., Sluijter J.P. (2015). Gap junctional protein Cx43 is involved in the communication between extracellular vesicles and mammalian cells. Sci. Rep..

[20] Billaud M., Marthan R., Savineau J.P., Guibert C. (2009). Vascular smooth muscle modulates endothelial control of vasoreactivity via reactive oxygen species production through myoendothelial communications. PLoS ONE..

[21] Billaud M., Dahan D., Marthan R., Savineau J.P., Guibert C. (2011). Role of the gap junctions in the contractile response to agonists in pulmonary artery from two rat models of pulmonary hypertension. Respir. Res..

[22] Nakamura K., INAI T., Nakamura K., Shibata Y. (1999). Distribution of gap junction protein connexin 37 in smooth muscle cells of the rat trachea and pulmonary artery. Arch. Histol. Cytol..

[23] Li X., Simard J.M. (2001). Connexin45 gap junction channels in rat cerebral vascular smooth muscle cells. Am. J. Physiol. Heart Circ. Physiol..

[24] McNair A.J., Wilson K.S., Martin P.E., Welsh D.J., Dempsie Y. (2020). Connexin 43 plays a role in proliferation and migration of pulmonary arterial fibroblasts in response to hypoxia. Pulm. Circ..

[25] Yeh H.I., Rothery S., Dupont E., Coppen S.R., Severs N.J. (1998). Individual gap junction plaques contain multiple connexins in arterial endothelium. Circ. Res..

[26] Ko Y.S., Yeh H.I., Rothery S., Dupont E., Coppen S.R., Severs N.J. (1999). Connexin make-up of endothelial gap junctions in the rat pulmonary artery as revealed by immunoconfocal microscopy and triple-label immunogold electron microscopy. J. Histochem. Cytochem..

[27] Gairhe S., Bauer N.N., Gebb S.A., McMurtry I.F. (2011). Myoendothelial gap junctional signaling induces differentiation of pulmonary arterial smooth muscle cells. Am. J. Physiol. Lung Cell. Mol. Physiol..

[28] Bouvard C., Genet N., Phan C., Rode B., Thuillet R., Tu L., Robillard P., Campagnac M., Soleti R., De La Roque E.D. (2020). Connexin-43 is a promising target for pulmonary hypertension due to hypoxaemic lung disease. Eur. Respir. J..

[29] Kim J., Hwangbo C., Hu X., Kang Y., Papangeli I., Mehrotra D., Park H., Ju H., McLean D.L., Comhair S.A. (2015). Restoration of impaired endothelial myocyte enhancer factor 2 function rescues pulmonary arterial hypertension. Circulation.

[30] Kang Y., Kim J., Anderson J.P., Wu J., Gleim S.R., Kundu R.K., McLean D.L., Kim J.D., Park H., Jin S.W. (2013). Apelin-APJ signaling is a critical regulator of endothelial MEF2 activation in cardiovascular development. Circ. Res..

[31] Zhao L., Chen C.N., Hajji N., Oliver E., Cotroneo E., Wharton J., Wang D., Li M., McKinsey T.A., Stenmark K.R. (2012). Histone deacetylation inhibition in pulmonary hypertension: Therapeutic potential of valproic acid and suberoylanilide hydroxamic acid. Circulation.

[32] Si R., Zhang Q., Cabrera J.T., Zheng Q., Tsuji-Hosokawa A., Watanabe M., Hosokawa S., Xiong M., Jain P.P., Ashton A.W. (2020). Chronic Hypoxia Decreases Endothelial Connexin 40, Attenuates Endothelium-Dependent Hyperpolarization–Mediated Relaxation in Small Distal Pulmonary Arteries, and Leads to Pulmonary Hypertension. J. Am. Heart Assoc..

[33] Yang L., Yin N., Hu L., Fan H., Yu D., Zhang W., Wang S., Feng Y., Fan C., Cao F. (2014). Sildenefil increases connexin 40 in smooth muscle cells through activation of BMP pathways in pulmonary arterial hypertension. Int. J. Clin. Exp. Pathol..

[34] Welsh D.J., Scott P.H., Peacock A.J. (2006). p38 MAP kinase isoform activity and cell cycle regulators in the proliferative response of pulmonary and systemic artery fibroblasts to acute hypoxia. Pulm. Pharmacol. Ther..

[35] Htet M., Nally J.E., Shaw A., Foote B.E., Martin P.E., Dempsie Y. (2018). Connexin 43 plays a role in pulmonary vascular reactivity in mice. Int. J. Mol. Sci..

[36] Chen M., Liu Y., Yi D., Wei L., Li Y., Zhang L. (2014). Tanshinone IIA promotes pulmonary artery smooth muscle cell apoptosis in vitro by inhibiting the JAK2/STAT3 signaling pathway. Cell. Physiol. Biochem..

[37] Hoeper M.M., Pausch C., Grünig E., Klose H., Staehler G., Huscher D., Pittrow D., Olsson K.M., Vizza C.D., Gall H. (2020). Idiopathic pulmonary arterial hypertension phenotypes determined by cluster analysis from the COMPERA registry. J. Heart Lung Transplant..

[38] Morris H., Denver N., Gaw R., Labazi H., Mair K., MacLean M.R. (2021). Sex differences in pulmonary hypertension. Clin. Chest Med..

[39] Firestone G.L., Kapadia B.J. (2012). Minireview: Regulation of gap junction dynamics by nuclear hormone receptors and their ligands. Mol. Endocrinol..

[40] Geimonen E., Jiang W., Ali M., Fishman G.I., Garfield R.E., Andersen J. (1996). Activation of protein kinase C in human uterine smooth muscle induces connexin-43 gene transcription through an AP-1 site in the promoter sequence. J. Biol. Chem..

[41] Geimonen E., Boylston E., Royek A., Andersen J. (1998). Elevated connexin-43 expression in term human myometrium correlates with elevated c-Jun expression and is independent of myometrial estrogen receptors. J. Clin. Endocrinol. Metab..

[42] Ren J., Wang X.H., Wang G.C., Wu J.H. (2013). 17β Estradiol regulation of connexin 43-based gap junction and mechanosensitivity through classical estrogen receptor pathway in osteocyte-like MLO-Y4 cells. Bone.

[43] Chen C.C., Lin C.C., Lee T.M. (2010). 17β-estradiol decreases vulnerability to ventricular arrhythmias by preserving Connexin43 protein in infarcted rats. Eur. J. Pharmacol..

[44] Tsai C.F., Cheng Y.K., Lu D.Y., Wang S.L., Chang C.N., Chang P.C., Yeh W.L. (2018). Inhibition of estrogen receptor reduces connexin 43 expression in breast cancers. Toxicol. Appl. Pharmacol..

[45] Dempsie Y., Morecroft I., Welsh D.J., MacRitchie N.A., Herold N., Loughlin L., Nilsen M., Peacock A.J., Harmar A., Bader M. (2008). Converging evidence in support of the serotonin hypothesis of dexfenfluramine-induced pulmonary hypertension with novel transgenic mice. Circulation.

[46] Chester A.H., Yacoub M.H. (2014). The role of endothelin-1 in pulmonary arterial hypertension. Glob. Cardiol. Sci. Pract..

[47] Mondejar-Parreño G., Callejo M., Barreira B., Morales-Cano D., Esquivel-Ruiz S., Filice M., Moreno L., Cogolludo A., Perez-Vizcaino F. (2019). miR-1 induces endothelial dysfunction in rat pulmonary arteries. J. Physiol. Biochem..

[48] Mondejar-Parreño G., Callejo M., Barreira B., Morales-Cano D., Esquivel-Ruiz S., Moreno L., Cogolludo A., Perez-Vizcaino F. (2019). miR-1 is increased in pulmonary hypertension and downregulates Kv1. 5 channels in rat pulmonary arteries. J. Physiol..

[49] Liu Y., Li Y., Li J., Zuo X., Cao Q., Xie W., Wang H. (2021). Inhibiting miR-1 attenuates pulmonary arterial hypertension in rats. Mol. Med. Rep..

[50] Aaronson P.I., Robertson T.P., Knock G.A., Becker S., Lewis T.H., Snetkov V., Ward J.P. (2006). Hypoxic pulmonary vasoconstriction: Mechanisms and controversies. J. Physiol..

[51] Michelakis E.D., Thébaud B., Weir E.K., Archer S.L. (2004). Hypoxic pulmonary vasoconstriction: Redox regulation of O_2_-sensitive K^+^ channels by a mitochondrial O_2_-sensor in resistance artery smooth muscle cells. J. Mol. Cell. Cardiol..

[52] Weissmann N., Sommer N., Schermuly R.T., Ghofrani H.A., Seeger W., Grimminger F. (2006). Oxygen sensors in hypoxic pulmonary vasoconstriction. Cardiovasc. Res..

[53] Wang L., Yin J., Nickles H.T., Ranke H., Tabuchi A., Hoffmann J., Tabeling C., Barbosa-Sicard E., Chanson M., Kwak B.R. (2012). Hypoxic pulmonary vasoconstriction requires connexin 40–mediated endothelial signal conduction. J. Clin. Investig..

[54] Kizub I.V., Strielkov I.V., Shaifta Y., Becker S., Prieto-Lloret J., Snetkov V.A., Soloviev A.I., Aaronson P.I., Ward J.P. (2013). Gap junctions support the sustained phase of hypoxic pulmonary vasoconstriction by facilitating calcium sensitization. Cardiovasc. Res..

[55] Dempsie Y., MacLean M.R. (2008). Role of the serotonin transporter in pulmonary arterial hypertension. Expert Rev. Clin. Pharmacol..

[56] Gairhe S., Bauer N.N., Gebb S.A., McMurtry I.F. (2012). Serotonin passes through myoendothelial gap junctions to promote pulmonary arterial smooth muscle cell differentiation. Am. J. Physiol. Lung Cell. Mol. Physiol..

[57] Aasen T., Johnstone S., Vidal-Brime L., Lynn K.S., Koval M. (2018). Connexins: Synthesis, post-translational modifications, and trafficking in health and disease. Int. J. Mol. Sci..

[58] Johnstone S.R., Kroncke B.M., Straub A.C., Best A.K., Dunn C.A., Mitchell L.A., Peskova Y., Nakamoto R.K., Koval M., Lo C.W. (2012). MAPK phosphorylation of connexin 43 promotes binding of cyclin E and smooth muscle cell proliferation. Circ. Res..

[59] Good M.E., Nelson T.K., Simon A.M., Burt J.M. (2011). A functional channel is necessary for growth suppression by Cx37. J. Cell Sci..

[60] Good M.E., Ek-Vitorín J.F., Burt J.M. (2014). Structural determinants and proliferative consequences of connexin 37 hemichannel function in insulinoma cells. J. Biol. Chem..

[61] Morel S., Burnier L., Roatti A., Chassot A., Roth I., Sutter E., Galan K., Pfenniger A., Chanson M., Kwak B.R. (2010). Unexpected role for the human Cx37 C1019T polymorphism in tumour cell proliferation. Carcinogenesis.

[62] Han X.J., Zhang W.F., Wang Q., Li M., Zhang C.B., Yang Z.J., Tan R., Gan L., Zhang L., Lan X. (2021). HIF-1α promotes the proliferation and migration of pulmonary arterial smooth muscle cells via activation of Cx43. J. Cell Mol. Med..

[63] van der Velden H.M., Jongsma H.J. (2002). Cardiac gap junctions and connexins: Their role in atrial fibrillation and potential as therapeutic targets. Cardiovasc. Res..

[64] Chang L.T., Sun C.K., Sheu J.J., Chiang C.H., Youssef A.A., Lee F.Y., Wu C.J., Yip H.K. (2008). Cilostazol therapy attenuates monocrotaline-induced pulmonary arterial hypertension in rat model. Circ. J..

[65] Sun C.K., Lin Y.C., Yuen C.M., Chua S., Chang L.T., Sheu J.J., Lee F.Y., Fu M., Leu S., Yip H.K. (2012). Enhanced protection against pulmonary hypertension with sildenafil and endothelial progenitor cell in rats. Int. J. Cardiol..

[66] Lee F.Y., Lu H.I., Zhen Y.Y., Leu S., Chen Y.L., Tsai T.H., Chung S.Y., Chua S., Sheu J.J., Hsu S.Y. (2013). Benefit of combined therapy with nicorandil and colchicine in preventing monocrotaline-induced rat pulmonary arterial hypertension. Eur. J. Pharm. Sci..

[67] Sasano C., Honjo H., Takagishi Y., Uzzaman M., Emdad L., Shimizu A., Murata Y., Kamiya K., Kodama I. (2007). Internalization and dephosphorylation of connexin43 in hypertrophied right ventricles of rats with pulmonary hypertension. Circ. J..

[68] Tan X.Y., He J.G. (2009). The remodeling of connexin in the hypertrophied right ventricular in pulmonary arterial hypertension and the effect of a dual ET receptor antagonist (bosentan). Pathol. Res. Pract..

[69] Strauss B., Sassi Y., Bueno-Beti C., Ilkan Z., Raad N., Cacheux M., Bisserier M., Turnbull I.C., Kohlbrenner E., Hajjar R.J. (2019). Intra-tracheal gene delivery of aerosolized SERCA2a to the lung suppresses ventricular arrhythmias in a model of pulmonary arterial hypertension. J. Mol. Cell. Cardiol..

[70] Marsh S.R., Williams Z.J., Pridham K.J., Gourdie R.G. (2021). Peptidic Connexin43 Therapeutics in Cardiac Reparative Medicine. J. Cardiovasc. Dev. Dis..

[71] Naus C.C., Giaume C. (2016). Bridging the gap to therapeutic strategies based on connexin/pannexin biology. J. Transl. Med..

